# Prognostic value of noggin protein expression in patients with resected gastric cancer

**DOI:** 10.1186/s12885-021-08273-x

**Published:** 2021-05-17

**Authors:** Sang Hoon Chun, Eun Young Kim, Jung-Sook Yoon, Hye Sung Won, Kwangil Yim, Hye Won Hwang, Soon Auck Hong, Minho Lee, Su Lim Lee, Sung-Soo Kim, Der Sheng Sun, Yoon Ho Ko

**Affiliations:** 1 Division of Oncology, Department of Internal Medicine, College of Medicine, The Catholic University of Korea, Seoul, Republic of Korea; 2 Department of Surgery, College of Medicine, The Catholic University of Korea, Seoul, Republic of Korea; 3 Uijeongbu St. Mary’s Hospital Clinical Research Laboratory, The Catholic University of Korea, Seoul, Republic of Korea; 4 Department of Hospital Pathology, College of Medicine, The Catholic University of Korea, Seoul, Republic of Korea; 5 Department of Pathology, College of Medicine, Chung-Ang University, Seoul, Republic of Korea; 6 Department of Life Science, Dongguk University-Seoul, Goyang, Republic of Korea; 7 Department of Radiology, College of Medicine, The Catholic University of Korea, Seoul, Republic of Korea; 8 Department of Internal Medicine, Division of Gastroenterology, College of Medicine, The Catholic University of Korea, Seoul, Republic of Korea; 9 Cancer Research Institute, College of Medicine, The Catholic University of Korea, Seoul, Republic of Korea

**Keywords:** Gastric cancer, Noggin, RNA-binding protein for multiple splicing 2 (RBPMS2), Prognosis

## Abstract

**Background:**

Noggin and RNA-binding protein for multiple splicing 2 (RBPMS2) are known to regulate the expression of smooth muscle cells, endothelial cells, and osteoblasts. However, the prognostic role of combined Noggin and RBPMS2 expression in resected gastric cancer (GC) is unclear.

**Methods:**

A total of 163 patients with GC who underwent gastrectomy were included in this study. The expression of Noggin and RBPMS2 proteins in tumor cells at the tumor center and invasive front of resected GC was evaluated by immunohistochemistry, and in conjunction with clinicopathological parameters the patient survival was analyzed.

**Results:**

RBPMS2 protein expression was high at the tumor center (*n* = 86, 52.8%) and low at the invasive front (*n* = 69, 42.3%), while Noggin protein expression was high in both tumor center (*n* = 91, 55.8%) and the invasive front (*n* = 90, 55.2%). Noggin expression at the invasive front and tumor center was significantly decreased in advanced T stage, non-intestinal-type (invasive front, *P* = 0.008 and *P* <  0.001; tumor center lesion, *P* = 0.013 and *P* = 0.001). RBPMS2 expression at the invasive front was significantly decreased in non-intestinal-type and positive lymphatic invasion (P <  0.001 and P = 0.013). Multivariate analysis revealed that high Noggin protein expression of the invasive front was an independent prognostic factor for overall survival (hazard ratio [HR], 0.58; 95% confidence interval [CI]; 0.35–0.97, *P* <  0.036), but not at the tumor center (HR, 1.35; 95% CI; 0.81–2.26, *P* = 0.251).

**Conclusions:**

Our study indicates that high Noggin expression is a crucial prognostic factor for favorable outcomes in patients with resected GC.

**Supplementary Information:**

The online version contains supplementary material available at 10.1186/s12885-021-08273-x.

## Background

Gastric cancer (GC) is the fourth most common cancer and the third leading cause of cancer-related death globally [1]. Recently, early diagnosis and the development of surgical techniques have led to a significant improvement in clinical outcomes of patients with resectable GC. The 5-year survival rate of patients with early GC is over 90% [2]. Despite remarkable advances in targeted therapy for molecular targets, vascular endothelial growth factor receptor (VEGFR), and human epidermal growth factor receptor 2 (HER2) to improve patient survival in the setting of recurrent and metastatic GC, the prognosis of patients with recurrent and metastatic GC remains poor [3]. Thus, new molecular therapeutic targets and biomarkers are required to improve the survival of patients with GC.

Tumor stroma is composed of cancer-associated fibroblasts, immune cells, and other stromal cells around the cancer cells of extracellular matrix (ECM), which includes cell-adhesion molecules and ligands that play a role in tissue organization [4]. Besides, tumor-stroma interaction at the invasive front of the tumor represents a critical interface, where tumor progression and tumor cell dissemination occur due to the lack of cohesiveness, secretion of proteolytic enzymes, re-organization of the ECM, and increased cell proliferation [5].

Stromal gene expression or phenotype is one of the independent prognostic markers in various cancers including GC [6]. Recently, we identified a novel prognosis-associated four-gene signature comprising *NOGGIN* and RNA-binding protein for multiple splicing 2 (*RBPMS2*), cathepsin F (*CTSF*) and CUE domain containing 1 (*CUEDC1*), in resected GC using machine learning method [7] for effective stratification of 5-year survival outcomes. Noggin and RBPMS2 are known to regulate the expression of stromal cells, such as smooth muscle cells (SMCs), endothelial cells, and osteoblasts. Noggin protein is a glycosylated cysteine-knot protein that acts as an extracellular negative regulator of members in the transforming growth factor-beta superfamily that includes bone morphogenetic proteins (BMPs) [8]. Orthotopic expression of Noggin in endothelial cells results in the inhibition of cell migration and prevents the formation of endothelial cords in vitro, and Noggin inhibits angiogenesis even in the presence of pro-vasculogenic VEGF and fibroblastic growth factor-2 [9]. RBPMS2, a member of the RNA Recognition Motif family, is expressed in the vertebrate heart and gastrointestinal tract [10, 11]. It is also an early marker of gastrointestinal smooth muscle precursor cells [12]. Perturbations in RBP expression and function play an important role in cancer initiation and progression. Of note, the two proteins interact with each other and regulate the early development and plasticity of digestive SMCs by inhibiting the BMP pathway [13].

However, the expression pattern and prognostic values of Noggin and RBPMS2 in GC has yet to be determined. Furthermore, the expression of stroma-related proteins were not known to have different clinical implications between the tumor center and invasive regions in GC. Therefore, we evaluated the expression of Noggin and RBPMS2 proteins in the tumor center and invasive front of resected GC and compared their relationship with clinicopathological parameters and clinical outcomes.

## Methods

### Identification of molecular functions for *NOGGIN* and *RBPMS2* with GSE database

Genes predicting the prognosis of patients with GC were identified using the support vector machine algorithm for the microarray analysis of three publicly available gene expression profiles (GSE62254, GSE15459, and GSE15460) containing 822 samples of resected GC. To identify the molecular function enriched in *NOGGIN* and *RBPMS2* and positively correlated genes, we ranked genes based on their co-expression measured via linear-by-linear association test with *NOGGIN* and *RBPMS2*. Subsequently, the ranked gene lists were used to identify the enriched KEGG pathways using Gene Set Enrichment Analysis (GSEA) via GSEA-P [14].

### Patients and tissue samples

We performed a retrospective analysis of patients with GC at Uijeongbu St. Mary’s Hospital of the Catholic Medical Center between 2001 and 2005. The inclusion criteria were: pathologically confirmed adenocarcinoma, radical resection without preoperative radiation or chemotherapy, removal of at least 15 or more lymph nodes, and available tissue specimens. Neoadjuvant chemotherapy or radiotherapy was permitted. Pathological staging was based on the 7th edition of the American Joint Committee on Cancer staging criteria.

### Tissue microarray construction and immunohistochemistry

Tissue microarrays (TMA) were constructed for immunohistochemistry (IHC). The tissue cores (2 mm) were obtained from two representative paraffin block-embedded tumor regions in each case, containing invasive front, tumor cells or clusters at the perpendicularly deepest site of tumor invasion, and tumor center, the area that is equidistant for the tumor surface [15]. Briefly, the sections were deparaffinized and rehydrated using xylene and alcohol. Antigen retrieval was performed by heating the slides for 20 min in Tris-EDTA buffer (pH 9.0) and then blocking the endogenous peroxidase activity by quenching with 3% hydrogen peroxide in methanol for 10 min. The following steps in IHC were performed using R.T.U Vectastain Universal Quick kit (PK-7800; Vector Laboratories, Burlingame, CA) according to the manufacturer’s instructions. The sections were incubated with primary antibodies against rabbit polyclonal Noggin (1:100, catalogue number ab16054; Abcam, Cambridge, UK) and rabbit polyclonal RBPMS2 (1:50, catalogue number ab170777; Abcam) overnight at 4 °C. The antibody binding was visualized with ImmPACT DAB Peroxidase (HRP) Substrate kit (Vector, Burlingame, CA, USA). IHC staining of NOG and RBPMS2 was independently examined by two board certified pathologists (S.A.H. and K.Y.) who were blinded to clinicopathological variables. Discrepant cases between two observers were examined individually under a multiheaded microscope and discussed until an agreement was reached. The cytoplasmic expression of the tumor cells was considered positive. The staining intensity of the cytoplasmic expression at the invasive front and tumor center was evaluated, and graded semi-quantitatively as follows: 0, negative; 1, weak; 2, moderate; 3, strong. We considered a staining intensity of 0 and 1 as low and 2 and 3 as high.

### Statistical analysis

Independent Samples T-test was conducted to determine the association between protein expression and clinicopathological parameters. To study linear trends for the proportions of positive staining for the expression level of Noggin and RBPMS2 proteins, a crosstabulation was performed using chi-square tests. Overall survival (OS) was determined from the date of surgery to the date of death due to any cause or the last follow-up visit. Disease-free survival (DFS) was calculated from the date of surgery to the date of the first disease recurrence or last follow-up.

DFS and OS rates were measured using the Kaplan-Meier method, and statistical differences between the cumulative survival curves were evaluated using the log-rank test. Cox proportional hazards regression models were used to evaluate the significance of the prognostic factors. All variables with a *P*-value < 0.10 in the univariate analysis were included in the multivariate analysis. Survival rates and hazard ratio (HR) s are reported with 95% confidence intervals (CIs). A *P*-value of < 0.05 is considered statistically significant. The statistical analyses were performed using R statistical programming language version 3.4.1 (https://www.r-project.org).

## Results

### Role of *NOGGIN* and *RBPMS2* in stromal cell function

In the previous analysis of three publicly available gene expression profiles for GC, novel prognosis-associated four-gene signatures, such as *NOGGIN, RBPMS2, CTSF, and CUEDC1*, showed a moderate performance with an area under ROC curve (AUC) of 0.745 for the prediction of 5-year survival [7]. To identify the molecular pathways enriched in the co-expressed stroma-related genes, *NOGGIN* and *RBPMS2*, we conducted GSEA for KEGG pathways and identified the enriched pathways (Supplementary Fig. 1). Many pathways were common in both cases. “Focal adhesion” and “ECM receptor interaction” signatures were enriched and positively correlated with the expression of both *NOGGIN* and *RBPMS2*, respectively.

### Patients’ characteristics

A total of 163 paraffin blocks of tumor samples were prepared from patients who had undergone surgical gastrectomy. The clinical and pathological characteristics of the cohort are listed in Table 1. The patient cohort consisted of 117 males (71.8%) and 46 females (28.2%), with a median age of 69 (35–92) years. According to the pathological TNM staging criteria, 26 patients (16.0%) had stage I disease, 44 (27.0%) patients had stage II disease, 75 (46.0%) had stage III disease, and 18 (11.0%) had IV disease. One hundred five patients (64.4%) manifested regional lymph node metastases at the time of operation. The peritoneal seeding revealed 18 positive cases (11.0%). Adjuvant chemotherapy was performed 72 patients (44.2%) and there was no received adjuvant radiotherapy. During the median follow-up of 42.5 months (range, 0.1–121 months) after surgical resection, 103 (63.2%) died and 60 (36.8%) were alive at the last follow-up. Disease recurrence was observed in 66 cases (40.5%).

**Table 1:** Baseline clinicopathological characteristics of patients with gastric cancer (*n* = 163)

Variables	Data
Age, years, median (range)	69.0 (35–92)
≤ 70	93 (57.1)
> 70	70 (42.9)
Sex	
Male	117 (71.8)
Female	46 (28.2)
T stage	
T2	38 (23.3)
T3	47 (28.8)
T4	78 (47.9)
N stage	
N0	58 (35.6)
N1	22 (13.5)
N2	22 (13.5)
N3	61 (37.4)
Pathological stage	
I	26 (16.0)
II	44 (27.0)
III	75 (46.0)
IV	18 (11.0)
WHO differentiation	
Well/moderate	71 (43.6)
Poor	90 (55.2)
Lauren’s classification	
Intestinal type	78 (47.9)
Non-intestinal type^a^	85 (52.1)
Lymphatic invasion	
No	54 (33.1)
Yes	109 (66.9)
Vascular invasion	
No	135 (82.8)
Yes	28 (17.2)
R0 resection	
No	18 (11.0)
Yes	145 (89.0)
Adjuvant chemotherapy	
No	91 (55.8)
Yes	72 (44.2)
EBV positivity	
No	153 (93.9)
Yes	10 (6.1)
Peritoneal seeding	
Negative	145 (89.0)
Positive	18 (11.0)

Data are presented as number (%) unless otherwise indicated

^a^Included diffuse or mixed Lauren’s type

*EBV *Epstein–Barr virus

### Expression of noggin and RBPMS2 in normal gastric tissue compared with GC

**Fig. 1:**
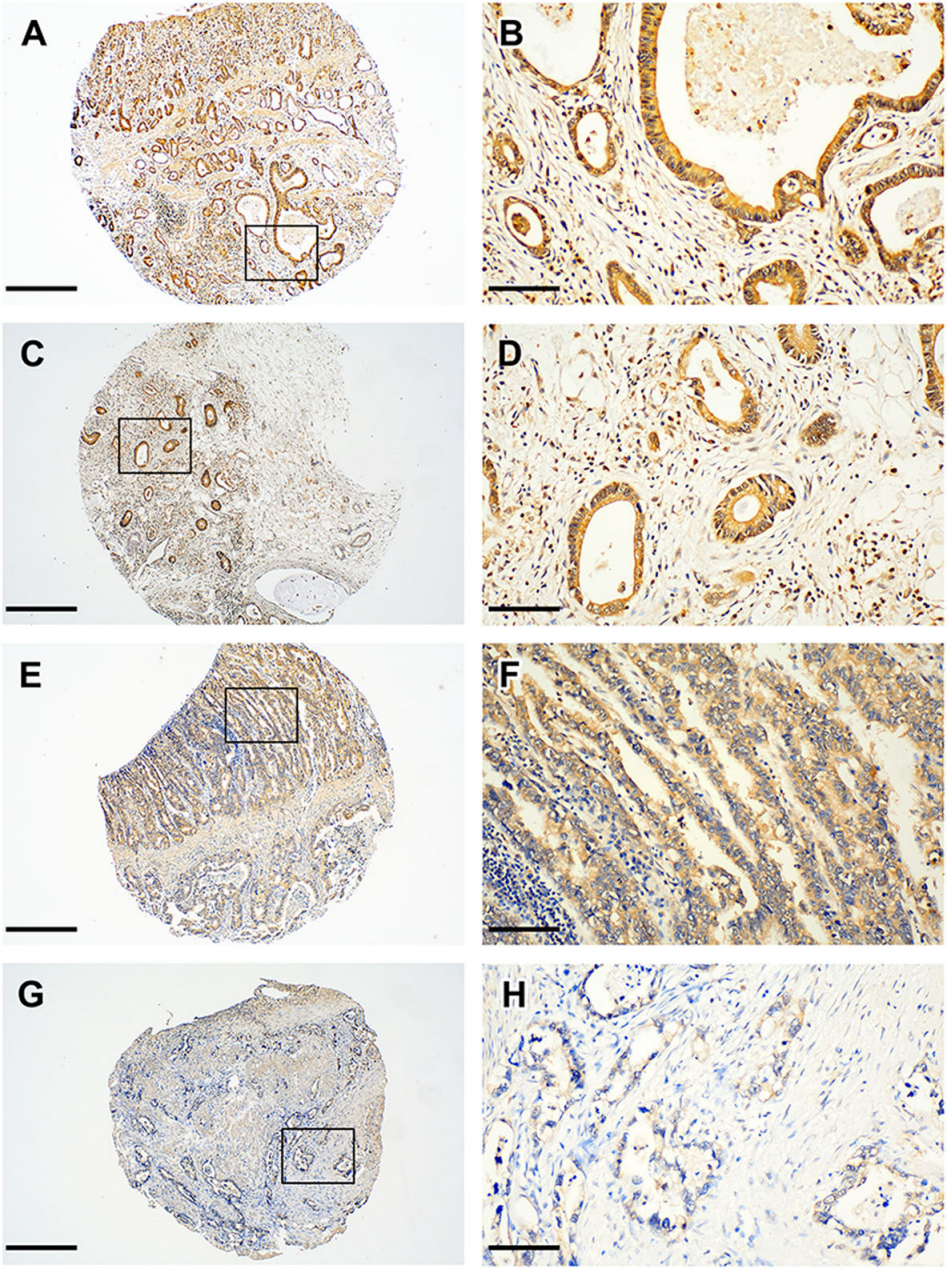
Representative immunohistochemical staining for Noggin and RBMPS2 expression in the paired cases of invasive front and tumor center of gastric cancer. High Noggin expression in tumor center [(**a** Original magnification ×40; scale bar, 500 μm) and (**b** Original magnification ×200; scale bar; 100 μm)] and deep invasive front [(**c** Original magnification × 40; scale bar, 500 μm)] and [(**d** Original magnification × 200; scale bar, 100 μm)] were detected; and high RBPMS2 expression in tumor center [(**e** Original magnification × 40; scale bar; 500 μm) and (**f** Original magnification × 200; scale bar; 100 μm)]; and low RBPMS2 expression in deep invasive front [(**g** Original magnification × 40; scale bar; 500 μm) and (**h** Original magnification × 200; scale bar; 100 μm)] were demonstrated. RBMPS2, RNA-binding protein for multiple splicing 2

In normal gastric tissues, noggin and RBPMS2 were weakly expressed in gastric parietal cells, while foveolar cells generally test negative for both proteins (Supplementary Fig. 2). Considering the invasive front and tumor center, the high expression of Noggin was similar in the invasive front (*n* = 90, 55.2%) and the tumor center (*n* = 91, 55.8%). High expression of Noggin was observed in 72 (61.3%) patients at both tumor center and invasive front (Fig. 1a-d). However, the high expression of RBPMS2 in tumor center (*n* = 86, 52.8%) was more frequent than in the invasive front (*n* = 69, 42.3%, Table 2, Supplementary Table 1). Among 31 cases with the discrepant expression of RBPMS2 between the tumor center and invasive front, high RBPMS2 expression in the tumor center and low in the invasive front accounted for 24 (77.4%) cases (Fig. 1e-h).

**Table 2:** Correlations between clinicopathological findings and Noggin or RBPMS2 protein expression in invasive front

Variables	Noggin (invasive)			RBPMS2 (invasive)		
	Low (*n* = 73)	High (n = 90)	*P*-value	Low (*n* = 94)	High (*n* = 69)	*P*-value
Pathological T stage			0.008			0.132
T2/T3	30 (35.3)	55 (64.7)		45 (52.9)	40 (47.1)	
T4	43 (55.1)	35 (44.9)		49 (62.8)	29 (37.2)	
Pathological N stage			0.070			0.051
N0	21 (36.2)	37 (63.8)		28 (48.3)	30 (51.7)	
N1-N3	52 (49.5)	53 (50.5)		66 (62.9)	39 (37.1)	
Lauren’s classification			< 0.001			< 0.001
Intestinal type	24 (30.8)	54 (69.2)		32 (41.0)	46 (59.0)	
Non-intestinal type^a^	49 (57.6)	36 (42.4)		62 (72.9)	23 (27.1)	
Lymphatic invasion			0.185			0.013
No	21 (38.9)	33 (61.1)		24 (44.4)	30 (56.6)	
Yes	52 (47.7)	57 (52.3)		70 (64.2)	39 (35.8)	
Vascular invasion			0.505			0.287
No	60 (44.4)	75 (55.6)		76 (56.3)	59 (43.7)	
Yes	13 (46.4)	15 (53.6)		18 (64.3)	10 (35.7)	
EBV positivity			0.250			0.425
No	67 (43.8)	86 (56.2)		89 (58.2)	64 (41.8)	
Yes	6 (60.0)	(40.0)		5 (50.0)	5 (50.0)	
Peritoneal seeding			0.234			0.141
Negative	63 (43.4)	82 (56.6)		81 (55.9)	64 (44.1)	
Positive	10 (55.6)	8 (44.4)		13 (72.2)	5 (27.8)	

Data are presented as number (%)

^a^Included diffuse or mixed Lauren’s type;

*RBPMS2* RNA-binding protein for multiple splicing 2, *EBV* Epstein–Barr virus

### Clinicopathological characteristics of resected gastric cancer patients according to noggin and RBPMS2 protein expression

The association between Noggin and RBPMS2 expression and clinicopathological features, including well-known prognostic factors such as pathologic TNM stage, lymphatic and vascular invasion, Lauren’s classification, Epstein–Barr virus (EBV) positivity and peritoneal seeding, was explored (Table 2 and Supplementary Table 1). Low Noggin expression at the invasive front and tumor center lesion was significantly correlated with advanced T stage and non-intestinal GC (invasive front, *P* = 0.008 and *P* <  0.001; tumor center lesion, *P* = 0.013 and *P* = 0.001, respectively). Low RBPMS2 protein expression at the invasive front was associated with non-intestinal type and positive lymphatic invasion (*P* <  0.001 and *P* = 0.013, respectively). Low expression of RBPMS2 protein in the tumor center was significantly correlated with the non-intestinal type and positive lymphatic invasion (*P* <  0.001 and *P* = 0.047). Further, the investigated proteins showed significant associations between Noggin and RBPMS2 proteins in the invasive front as well as the tumor center (*P* <  0.001, Table 3).

**Table 3:** Relationship between the expression patterns of NOG and RBPMS2 proteins

		Noggin (invasive)			RBPMS2 (invasive)			Noggin (center)		
		Low	High	Total	Low	High	Total	Low	High	Total
RBPMS2 (invasive)	Low	62 (84.9%)	32 (35.6%)	94 (57.7%)						
	High	11 (15.1%)	58 (64.4%)	69 (42.3%)						
	Total	73,(100.0%)	90(100.0%)	163,(100.0%)						
	*p*-value	< 0.001								
Noggin (center)	Low	54 (74.0%)	18 (20.0%)	72 (44.2%)	53 (56.4%)	19 (27.5%)	72 (44.2%)			
	High	19 (26.0%)	72 (80.0%)	91 (55.8%)	41 (43.6%)	50 (72.5%)	91 (55.8%)			
	Total	73(100.0%)	90(100.0%)	163(100.0%)	94(100.0%)	69(100.0%)	163(100.0%)			
	*p-*value	< 0.001			< 0.001					
RBPMS2 (center)	Low	51 (69.9%)	26 (28.9%)	77 (47.2%)	70 (74.5%)	7 (10.1%)	78 (47.2%)	48 (66.7%)	29 (31.9%)	77 (47.2%)
	High	22 (30.1%)	64 (71.1%)	86 (52.8%)	24 (25.5%)	62 (89.9%)	86 (52.8%)	24 (33.3%)	62 (68.1%)	86 (52.8%)
	Total	73(100.0%)	90(100.0%)	163(100.0%)	94(100.0%)	69(100.0%)	163(100.0%)	72(100.0%)	91(100.0%)	163(100.0%)
	*p-*value	< 0.001			< 0.001			< 0.001		

Used by chi-square test

*RBPMS2* RNA-binding protein for multiple splicing 2

### Prognostic value of noggin and RBPMS2 expression in resected GC

**Fig. 2:**
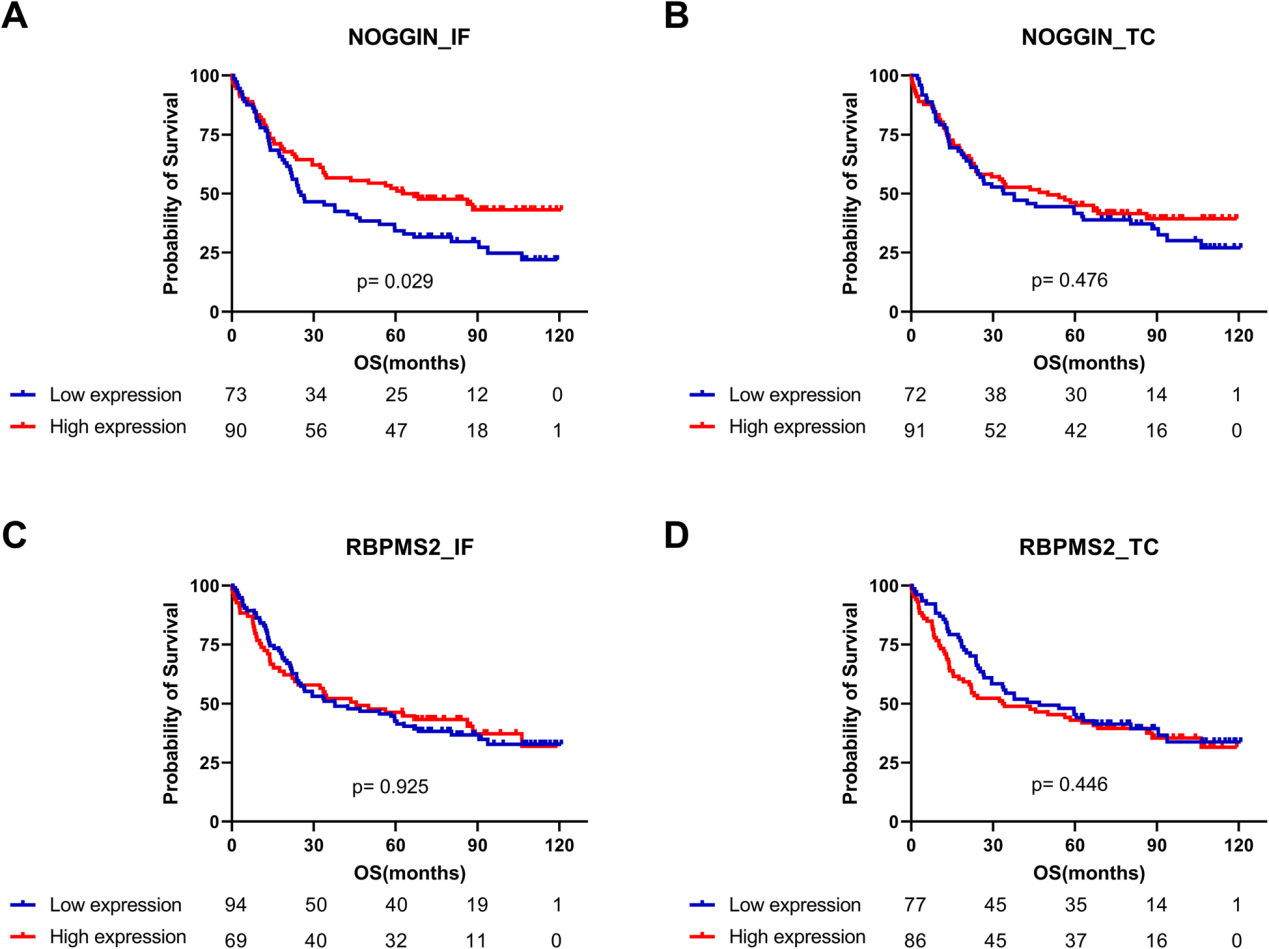
Kaplan-Meier survival curves for overall survival based on Noggin and RBPMS2 protein expression. Noggin protein expression at the invasive front (**a**) and tumor center (**b**); RBPMS2 expression in the invasive front (**c**) and tumor center (**d**). OS, overall survival; RBMPS2, RNA-binding protein for multiple splicing 2; IF, invasive front; TC, tumor center

**Fig. 3:**
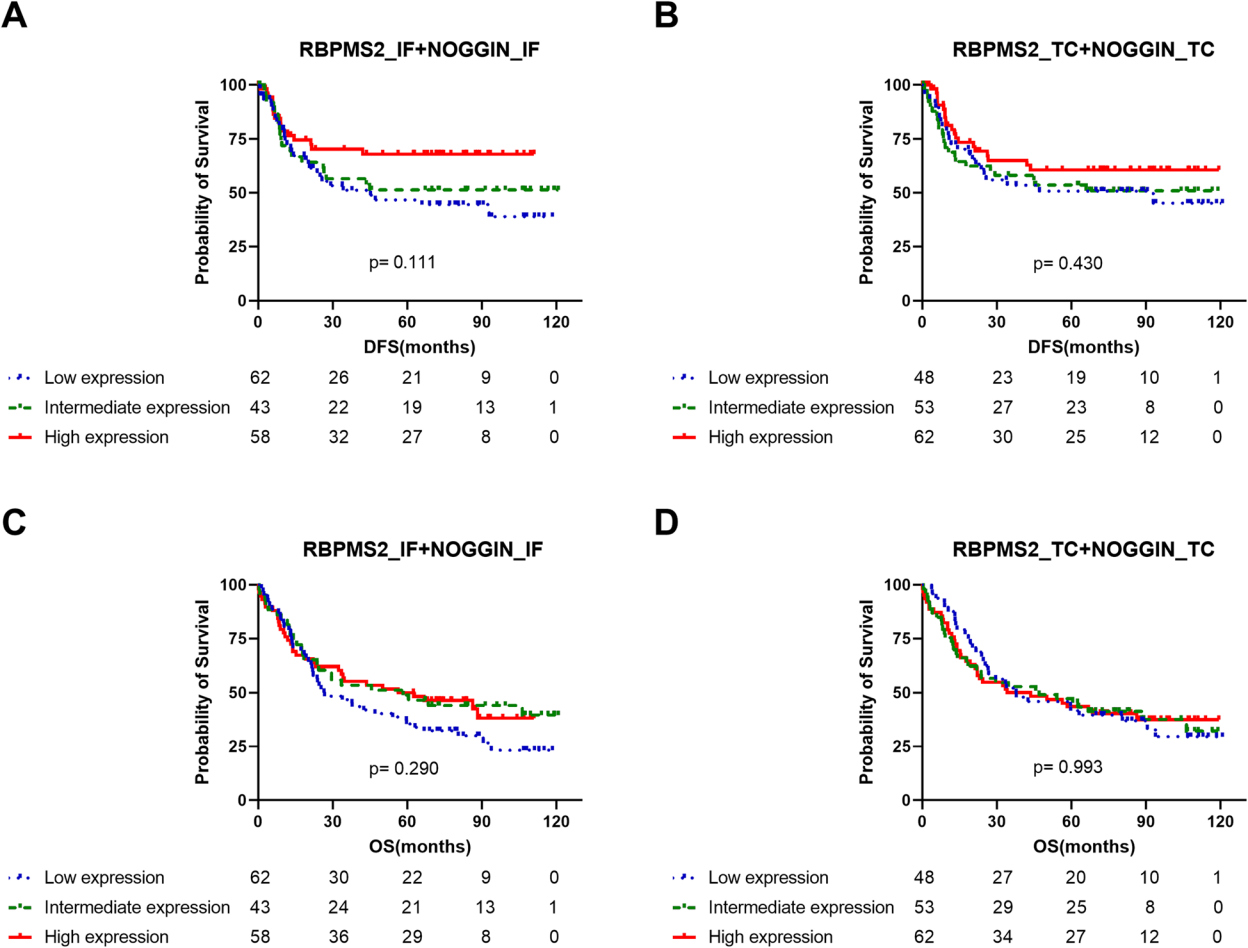
Kaplan-Meier survival curves for disease-free survival and overall survival by combined expression of Noggin and RBPMS2 proteins. Noggin and RBPMS2 expression of the invasive front (**a**) and the tumor center (**b**) by DFS; Noggin and RBPMS2 expression in the invasive front (**c**) and tumor center lesion (**d**) by OS. (Low expression = Low Noggin and RBPMS2 expression; Intermediate expression = One of them is low; High expression = High Noggin and RBPMS2 expression). DFS, disease-free survival; OS, overall survival; RBMPS2, RNA-binding protein for multiple splicing 2; IF, invasive front; TC, tumor center

We assessed the correlation between Noggin and RBPMS2 expression, and clinical prognosis using Kaplan-Meier curves with log-rank test. The elevated expression of Noggin at the invasive front was significantly associated with longer OS (*P* = 0.029, Fig. 2a), but not at the tumor center lesion (*P* = 0.476, Fig. 2b). No differences in OS were found between patients with high and low RBPMS2 expression in the invasive front (*P* = 0.925, Fig. 2c) and tumor center (*P* = 0.446, Fig. 2d). Moreover, the expression of stromal proteins was strongly and significantly correlated with each other (Table 3). Thus, the 165 cases in the study were divided into 3 groups according to the number of proteins acquired, and a survival analysis was also performed (Fig. 3). Patients with high Noggin and RBPMS2 expression at the invasive front demonstrated a marginal significance for longer DFS compared with other groups (*P* = 0.111, Fig. 3a), but not in OS (*P* = 0.290, Fig. 3c).

The factors affecting OS were further analyzed using Cox proportional hazards regression method. The univariate Cox proportional hazard ratios for OS were calculated. Univariate analysis revealed that the following factors significantly worsened the OS: old age (*P* <  0 .001), advanced pathologic T stage (*P* <  0.001), lymph node metastasis (*P* <  0.001), lymphatic invasion (*P* <  0.001), vascular invasion (*P* = 0.030), no R0 resection (*P* <  0.001), and peritoneal seeding positivity (*P* <  0.001). High Noggin protein expression and EBV positivity at the invasive front were significantly correlated with better OS (*P* = 0.030 and *P* = 0.020), but not the high Noggin protein expression in tumor center lesions and RBPMS2 protein expression at the invasive front and tumor center (Table 4). Results of multivariate Cox proportional hazards model showed that the high Noggin protein expression in invasive front (HR = 0.58; 95% CI, 0.35–0.97; *P* = 0.036) was a significant independent prognostic indicator for OS (Table 4). However, Noggin protein expression at the invasive front displayed a trend toward DFS in univariate analysis (*P* = 0.054), without reaching statistical significance in multivariate analysis (Supplementary Table 2 and Supplementary Fig. 3).

**Table 4:** Univariate and multivariate analyses using Cox proportional-hazards model for all patients based on overall survival

Variables	Univariate			Multivariate		
	HR	95% CI	*P*-value	HR	95% CI	*P*-value
Age **(> 70** vs. ≤ 70)	2.203	1.497–3.270	< 0.001	2.562	1.698–3.852	< 0.001
Sex (**female** vs. male)	1.172	0.771–1.793	0.460			0.404
T stage (**T4** vs. T2/3)	2.772	1.850–4.144	< 0.001	1.560	0.940–2.581	0.085
N stage (**N1–3** vs. N0)	3.724	2.292–6.041	< 0.001	3.007	1.663–5.461	< 0.001
Lauren’s classification (**non-intestinal** vs. intestinal)	1.334	0.898–1.962	0.154			0.675
Lymphatic invasion (**yes** vs. no)	3.141	1.921–5.143	< 0.001	1.481	0.790–2.789	0.219
Vascular invasion (**yes** vs. no)	1.713	1.054–2.761	0.030	1.209	0.734–2.021	0.455
R0 resection (**no** vs. yes)	2.662	1.552–4.580	< 0.001	0.631	0.181–2.171	0.461
High Noggin, invasive front (**yes** vs. no)	0.653	0.444–0.958	0.030	0.583	0.351–0.969	0.036
High Noggin, center lesion (**yes** vs. no)	0.867	0.591–1.283	0.481			
High RBPMS2, invasive front (**yes** vs. no)	0.983	0.655–1.449	0.925			
High RBPMS2, center lesion (**yes** vs. no)	1.162	0.792–1.710	0.446			
EBV positivity (**yes** vs. no)	0.188	0.046–0.768	0.020	0.240	0.058–0.992	0.049
Peritoneal seeding (**positive** vs. negative)	3.102	1.549–4.584	< 0.001	2.065	0.588–7.231	0.256

*HR* Hazard ratio, *CI* Confidence interval, *RBPMS2* RNA-binding protein for multiple splicing 2, *EBV* Epstein–Barr virus

## Discussion

The prognostic value of stroma-related proteins, Noggin and RBPMS2, expressed in the tumor center or invasive front, is complex and debatable. Previous reports also showed an ambiguous role of Noggin and RBPMS2 expression in GC [16, 17]. In this study, we found that low Noggin protein expression at the invasive front of GC was more frequent in the group at an advanced T stage and in the non-intestinal Lauren’s subtype, suggesting that the high Noggin expression was associated with a favorable clinical outcome in patients with resected GC. Its prognostic effect appears to be independent of established clinicopathological factors. To the best of our knowledge, this is the first report focusing on the clinical significance of Noggin at the invasive front of tumors in patients with resected GC.

The *NOGGIN* gene resides on chromosome 17q22, and is generally deleted in human cancers. It encodes a secreted polypeptide called Noggin. Noggin, a BMP antagonist, plays an essential role in bone formation and homeostasis, organogenesis, carcinogenesis, and bone metastasis [8, 18–20]. The normal stomach tissues also exhibit moderate expression of Noggin, thus indicating its important role for BMP signaling pathway in the normal stomach [21]. Although the function of BMP signaling in tumorigenesis and tumor progression remains controversial, the overexpression of Noggin leads to decreased tumor size and reduced bone metastatic tumor growth in prostate cancer and lung cancer models [22, 23]. The tumor-suppressive function of *NOGGIN* is mediated by the inhibition of EMT-like transition [24], inhibition of Wnt signaling pathway [25] as well as the inhibition of BMP signaling [8, 20].

The growth of normal human gastric epithelial cells requires Noggin, EGF, R-spondin1, and Wnt3a [26]. Consistent with these findings, in this study, we observed that Noggin protein expression at the invasive front was more frequently upregulated in intestinal-type (68.4%) than in mixed or diffuse GC (43.0%) under Lauren’s classification (*P* <  0.001), suggesting its role to maintain gastric gland morphology. On the other hand, our results suggest the negative prognostic effect of Noggin protein expression in the patients with resected GC. Consistent with these results, several previous studies showed that low levels of Noggin protein in combination with high BMP expression are associated with poor prognosis in esophageal carcinoma and increased metastasis in prostate and esophageal cancer [27, 28]. In contrast, Sun et al., in a study of 321 patients with GC and in vitro experiments, demonstrated that Noggin is associated with a poor prognosis of GC by promoting the proliferation of GC cells via the upregulation of epidermal growth factor receptor (EGFR). However, the different functions of Noggin protein were observed between intestinal- and diffuse type-GC cells, and the authors did not analyze its prognostic role according to Lauren’s classification in the patients with GC [29]. In addition, the expression of BMPs, one of the targets of Noggin, is upregulated and correlated with poor survival of GC patients. In a study of 178 gastric tumor biopsies, the expression of BMP-2 and Matrix metallopeptidase (MMP)-9 showed a significant positive correlation with lymph node metastasis and a poor prognosis. The BMP-2 signaling pathway enhances tumor metastasis in GC via sequential activation of the PI3K/AKT or MAPK pathway activation [30]. Further, we previously reported the prognostic role of Wnt antagonist, dickkopf1 (DKK1) in the same patient population, indicating that the high DKK1 expression, regardless of ß-catenin positivity, is an important prognostic factor for predicting tumor recurrence and survival in resected GC patients [31]. Interestingly, DKK1 and Noggin functionally cooperate in the organization of mammalian head [32], and mediate bone metastasis in patients with solid cancer [33]. These findings suggest that Noggin in conjunction with Wnt antagonist DKK1 may play a crucial role in bone metastasis in patients with diffuse-type GC and serve as the candidate biomarker for adjuvant therapy of bone metastasis [34].

RBPMS family is generally represented by two paralogs in vertebrates, RBPMS and RBPMS2 [35], which shuttle between nuclear and cytoplasmic fractions. RBPMS2 mediates the development and plasticity of gastrointestinal smooth muscle precursor cells [12] and is an early marker of gastrointestinal smooth muscle precursor cells [36], which positively regulates mRNA expression of Noggin. Ectopic expression of RBPMS2 in differentiated digestive smooth muscle precursor cells hinders their ability to contract and induces their proliferation leading to dedifferentiation, demonstrating that RBPMS2 expression is tightly regulated to avoid dedifferentiation of SMCs. In a previous report of GIST, RBPMS2 was upregulated in GISTs compared with normal adult gastrointestinal tissues, and its expression was higher in high-risk than in low-risk GIST specimens [37]. However, the increased expression of RBPMS2 has been correlated with favorable clinical outcomes in pancreatic cancer [38], which was similar to our data. Thus, a further study is still needed to elucidate the clinical role of RBPMS2 in GC.

Interestingly, the expression of the Noggin protein at the invasive front is strongly correlated with prognostic factors, compared with those at the tumor center. Invasive cancer is a complex process that is related to cell attachment, matrix dissociation, migration, and tumor cell proliferation [39]. Thus, the invasive front of tumor cells is the most important area involved in prognosis and key molecular mechanisms in tumors aggravated via proliferation, angiogenesis, and loss of epithelial differentiation [5]. Previous studies also reported a discrepancy in protein expression between invasive front and tumor center based on prognostic or clinicopathological parameters [40–43]. In line with these findings, our data demonstrated that the expression of Noggin in the invasive front is a more representative parameter for prognostic evaluation of those proteins in advanced GC.

There are some limitations to our study. Because of the retrospective nature of this study, it might not reflect the biological properties of the entire population with GC. The investigated protein expression in the tumor center and the invasive front was compared via TMA instead of whole tumor sections. However, to compensate for the limitations of TMA, the tumor tissue was obtained in the most representative portion. Further, the differences in the interpretation of IHC staining method, tissue aging effects, staining techniques, and differences in enzyme antibodies used between studies contribute to the study limitations. Despite our limitations, we included a homogenous population that underwent standard surgical treatment for GC and was followed-up long-term.

## Conclusions

In surgically resected GC, Noggin protein expression was strongly correlated with RBPMS2 expression. Besides, high Noggin expression in the invasive front of the tumor was independently and significantly associated with prognosis. Taken together, these results suggest that Noggin may act as a prognostic biomarker in patients with resected GC.

## Supplementary Information

**Additional file 1: Supplementary Figure 1**. The molecular function of Noggin and RBPMS2. Gene set enrichment analysis (GSEA) of positively correlated genes with Noggin and RBPMS2 in GSE62254, GSE15459, and GSE15460 datasets showed “Focal adhesion” and “ECM receptor interaction” signatures, which were enriched and positively correlated with both Noggin and RBPMS2 expression, respectively (A). KEGG pathway analysis of positively correlated genes with Noggin and RBPMS2 associated with the GO terms shown on the left side (B). Normalized Enrichment Scores are represented by the bars, where the adjusted *p*-values (familywise error rate, FWER) ≤ 0.05 are presented with a green bar or FWER > 0.05 with a red bar. **Supplementary Figure 2**. Immunohistochemical staining of noggin (A, original magnification × 1 200; scale bar, 100 μm) and RBPMS2 (B, original magnification × 200; scale bar, 100 μm) in normal gastric mucosa. Foveolar cells are negative for noggin and RBPMS2, while both proteins are weakly expressed in parietal cells. **Supplementary Figure 3**. Kaplan-Meier survival curves for disease-free survival were plotted according to Noggin and RBPMS2 protein expression. Noggin protein expression at the invasive front (A) and tumor center (B); RBPMS2 expression in the invasive front (C) and tumor center (D). DFS, disease-free survival; RBMPS2, RNA-binding protein for multiple splicing 2; IF, invasive front; TC, tumor center.

**Additional file 2: Supplementary Table 1** Clinicopathological factors and association according to RBPMS2 and NOG expression in tumor center lesions. **Supplementary Table 2** Univariate and multivariate analyses of disease-free survival in all patients using Cox proportional-hazards model.

## Data Availability

The datasets used and/or analysed during the current study are available from the corresponding author on reasonable request.

## Abbreviations

AUC: Area under ROC curve
BMPs: Bone morphogenetic proteins
CI: Confidence interval
CTSF: Cathepsin F
CUEDC1: CUE domain containing 1
DFS: Disease free survival
DKK1: Dickkopf1
EBV: Epstein–Barr virus
ECM: Extracellular matrix
EGFR: Epidermal growth factor receptor
GC: Gastric cancer
GSEA: Gene Set Enrichment Analysis
HER2: Human epidermal growth factor receptor 2
HR: Hazard ratio
IHC: Immunohistochemistry
MMP: Matrix metallopeptidase
OS: Overall survival
RBPMS2: RNA-binding protein for multiple splicing 2
SMCs: Smooth muscle cells
TMA: Tissue microarrays
VEGFR: Vascular endothelial growth factor receptor
