# Supplementary figures and images for: Prognostic value of noggin protein expression in patients with resected gastric cancer
Source: BMC Cancer. 2021 May 17;21:558. doi: 10.1186/s12885-021-08273-x (PMC8130398; doi:10.1186/s12885-021-08273-x)

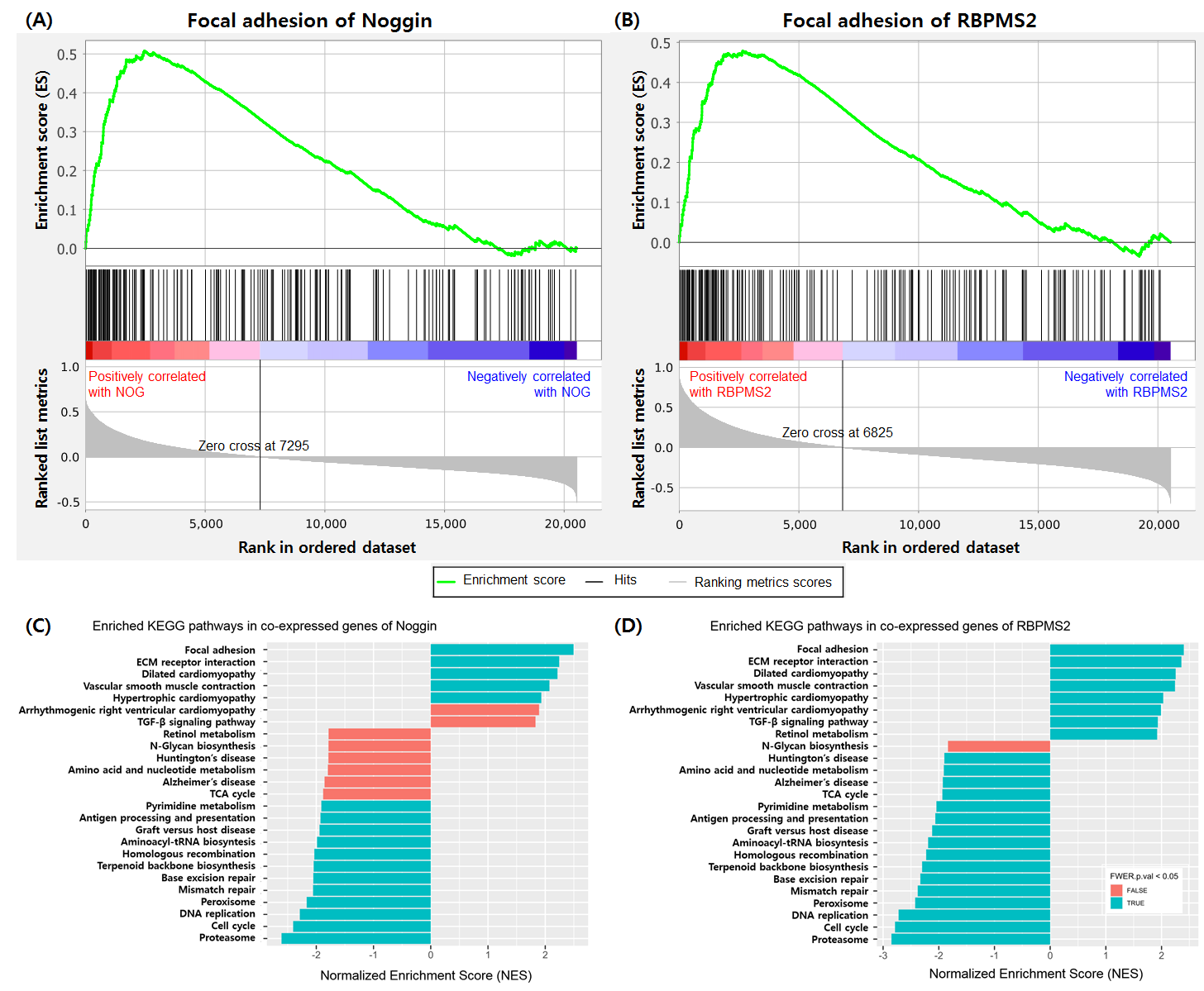

Supplement: Supplementary file 1 — Additional file 1: Supplementary Figure 1. The molecular function of Noggin and RBPMS2. Gene set enrichment analysis (GSEA) of positively correlated genes with Noggin and RBPMS2 in GSE62254, GSE15459, and GSE15460 datasets showed “Focal adhesion” and “ECM receptor interaction” signatures, which were enriched and positively correlated with both Noggin and RBPMS2 expression, respectively (A). KEGG pathway analysis of positively correlated genes with Noggin and RBPMS2 associated with the GO terms shown on the left side (B). Normalized Enrichment Scores are represented by the bars, where the adjusted p-values (familywise error rate, FWER) ≤ 0.05 are presented with a green bar or FWER > 0.05 with a red bar. Supplementary Figure 2. Immunohistochemical staining of noggin (A, original magnification × 1 200; scale bar, 100 μm) and RBPMS2 (B, original magnification × 200; scale bar, 100 μm) in normal gastric mucosa. Foveolar cells are negative for noggin and RBPMS2, while both proteins are weakly expressed in parietal cells. Supplementary Figure 3. Kaplan-Meier survival curves for disease-free survival were plotted according to Noggin and RBPMS2 protein expression. Noggin protein expression at the invasive front (A) and tumor center (B); RBPMS2 expression in the invasive front (C) and tumor center (D). DFS, disease-free survival; RBMPS2, RNA-binding protein for multiple splicing 2; IF, invasive front; TC, tumor center. [file 12885_2021_8273_MOESM1_ESM.zip › Supplementary Figure 1.tif]

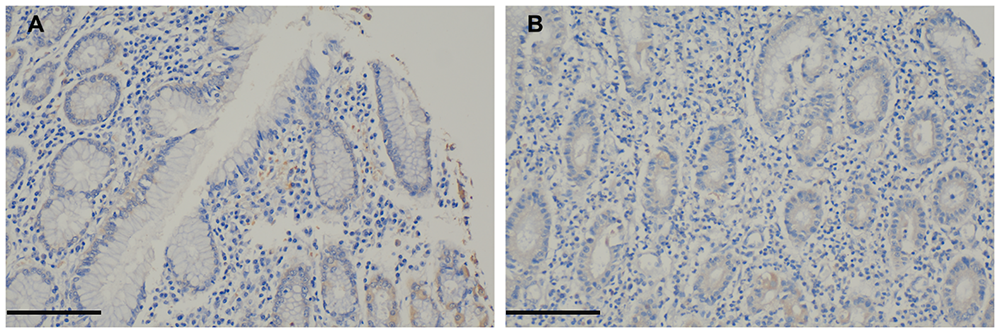

Supplement: Supplementary file 1 — Additional file 1: Supplementary Figure 1. The molecular function of Noggin and RBPMS2. Gene set enrichment analysis (GSEA) of positively correlated genes with Noggin and RBPMS2 in GSE62254, GSE15459, and GSE15460 datasets showed “Focal adhesion” and “ECM receptor interaction” signatures, which were enriched and positively correlated with both Noggin and RBPMS2 expression, respectively (A). KEGG pathway analysis of positively correlated genes with Noggin and RBPMS2 associated with the GO terms shown on the left side (B). Normalized Enrichment Scores are represented by the bars, where the adjusted p-values (familywise error rate, FWER) ≤ 0.05 are presented with a green bar or FWER > 0.05 with a red bar. Supplementary Figure 2. Immunohistochemical staining of noggin (A, original magnification × 1 200; scale bar, 100 μm) and RBPMS2 (B, original magnification × 200; scale bar, 100 μm) in normal gastric mucosa. Foveolar cells are negative for noggin and RBPMS2, while both proteins are weakly expressed in parietal cells. Supplementary Figure 3. Kaplan-Meier survival curves for disease-free survival were plotted according to Noggin and RBPMS2 protein expression. Noggin protein expression at the invasive front (A) and tumor center (B); RBPMS2 expression in the invasive front (C) and tumor center (D). DFS, disease-free survival; RBMPS2, RNA-binding protein for multiple splicing 2; IF, invasive front; TC, tumor center. [file 12885_2021_8273_MOESM1_ESM.zip › Supplementary Figure 2_revised.tif]
